# A Novel Fuzzy Expert System for the Identification of Severity of Carpal Tunnel Syndrome

**DOI:** 10.1155/2013/846780

**Published:** 2013-09-03

**Authors:** Reeda Kunhimangalam, Sujith Ovallath, Paul K. Joseph

**Affiliations:** ^1^National Institute of Technology, Calicut (NITC), Kozhikode, Kerala 673601, India; ^2^Department of Neurology, Kannur Medical College, Anjarakandy, Kannur, Kerala 670612, India

## Abstract

The diagnosis of carpal tunnel syndrome, a peripheral nerve disorder, at the earliest possible stage is very crucial because if left untreated it may cause permanent nerve damage reducing the chances of successful treatment. Here a novel Fuzzy Expert System designed using MATLAB is proposed for identification of severity of CTS. The data used were the nerve conduction study data obtained from Kannur Medical College, India. It consists of thirteen input fields, which include the clinical values of the diagnostic test and the clinical symptoms, and the output field gives the disease severity. The results obtained match with the expert's opinion with 98.4% accuracy and high degrees of sensitivity and specificity. Since quantification of the intensity of CTS is a crucial step in the electrodiagnostic procedure and is important for defining prognosis and therapeutic measures, such an expert system can be of immense use in those regions where the service of such specialists may not be readily available. It may also prove useful in combination with other systems in providing diagnostic and predictive medical opinions and can add value if introduced into the routine clinical consultations to arrive at the most accurate medical diagnosis in a timely manner.

## 1. Introduction

Carpal tunnel syndrome (CTS) is an entrapment type neuropathy due to the compression/entrapment of the median nerve of the wrist passing through the carpal tunnel [1]. The symptoms include weakness, numbness, parasthesia, and in some cases pain [2]. Nerve conduction study (NCS) is a fundamental component of electrodiagnostic evaluation, providing valuable quantitative and qualitative understanding of neuromuscular functions, especially the ability of electrical conduction of the motor and sensory nerves. NCS can be used for the confirmation of CTS as well as the quantification of the disease severity [3], thereby aiding in treatment decisions. Medication for one to two months is reasonable in mild to moderate CTS. Worsening or lasting clinical symptoms in spite of conservative treatment and clearly abnormal electro-diagnostic studies clearly indicate severe CTS which may have to be referred for surgical evaluation [4, 5]. The diagnosis at the earliest possible stage is very essential since long-standing CTS may cause permanent nerve damage and generally reduces the chances of successful surgical treatment [6, 7]. Surgery to correct CTS has a high success rate (about 90%) and most people relieved of their CTS symptoms with conservative/surgical management have minimal residual nerve damage. The diagnosis is usually made by a specialist neurologist, and this task involves basic symptoms elicitation and analysis of the NCS data using a combination of the patient's case history, current symptoms, and various electrophysiological findings [8].

The use of mathematical sciences, engineering principles, and computer technology in the diagnosis and treatment of various illnesses has highly increased nowadays. In spite of being highly complex and uncertain, intelligent systems such as fuzzy logic, artificial neural networks, and genetic algorithm have been widely employed in the field of medicine [9]. Fuzzy logic, a multivalued logic similar to human thinking and interpretation, is highly suitable and applicable for developing knowledge-based systems in medicine for interpretation of medical findings, diagnosis and treatment selection [10, 11]. A Fuzzy Expert System (FES) is a type of rule-based form of artificial intelligence using a collection of membership functions and rules to reason about data [12]. Our aim was to develop such an FES using fuzzy logic controller which could diagnose the stage of CTS, from the clinical symptoms and the NCS data, and thereby help the clinician in deciding the treatment options as soon as possible. The reason of choosing fuzzy logic for the development of such a system lies in the fact that fuzziness, described as the vagueness in the description of events or phenomena, is generally found wherever human decisions, judgment, or evaluation plays an important role. Diagnosis or classification of a disease is often done by the specialist using a set of rules and the designed system involves the collection of these rules, together with an inference engine for evaluating the rule base for a given set of inputs. This method allows imprecision in the user inputs as well as in the rule base specification. Fuzzy logic mimics the cognitive decision making ability of the specialist and enables the less experienced junior doctors to arrive at a better diagnosis as it keeps the expert knowledge in an intelligent system to be used efficiently by others. So such an expert system can be of immense use in those areas where the service of such specialists may not be available and its use is recommended to shorten the time and improve the accuracy of diagnosis in patients with suspected CTS [13, 14].

## 2. Materials and Methods

### 2.1. Database Description

In the above study we had used 135 NCS data obtained from the electronic medical records of the Kannur Medical College, Kerala, India, to check the accuracy of the developed FES. Out of the 135 cases, 36 were normal cases, who had normal NCS values and had no electro-physiological evidence of CTS, 56 were patients suffering from mild to moderate CTS, and the remaining were having severe CTS symptoms. All the patients selected were having isolated carpal tunnel syndrome and those with any pre existing nerve conditions were excluded. The cases with abnormal ulnar studies at the wrist were excluded to avoid other types of neuropathy being misdiagnosed as CTS. The ethical committee approval was obtained. The present study used the following median nerve and ulnar nerve measures: median motor and sensory latencies, median motor and sensory velocities, ulnar motor and sensory latencies, and ulnar motor and sensory velocities [15]. The attributes of the data used are given in Table 1.

The following criteria were applied for identifying the presence of CTS:

- median motor latency greater than 4.4 ms,
- median sensory latency greater than 3.84 ms,
- median motor and sensory velocities less than 50 m/sec,
- ulnar motor and sensory values will be normal (latency: 2.54 ± .29 ms, velocity: 55.7 ± 4.9 m/sec).

Higher values of median latencies and lower values of median NCVs indicate an increase in the severity of the disease. The ulnar values are unaffected; that is, they remain normal. If the ulnar values are abnormal it is an indication of neuropathy, another peripheral nerve disorder [16, 17].

#### 2.1.1. Procedure of NCS

NCS was performed using the standard techniques with surface electrode recording on both hands of each subject using constant current stimulator. The median motor nerve at the wrist was stimulated to obtain the median motor nerve data; the recording was done over the abductor pollicis brevis muscle. The ulnar motor data was obtained by stimulating the ulnar nerve at the wrist, below the elbow, and above the elbow and recording over the abductor digiti minimi muscle. Sensory responses were obtained by applying stimulation at the wrist and recording from the index finger to get the median data and the little finger to get the ulnar data.

#### 2.1.2. Design of Fuzzy Expert System

**Figure 1:**
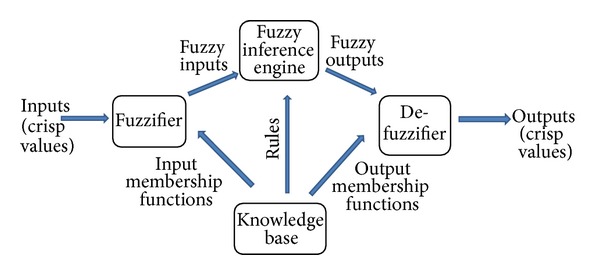
Outline of a basic Fuzzy Expert System.

 An expert system is a computer program that helps in solving problems demanding substantial human expertness by using explicitly exhibited domain knowledge and computational decision procedures. These are designed to make available some of the skills of an expert to nonexperts, as they attempt to imitate the thinking patterns and logical decisions of an expert. The FES makes use of the theory of fuzzy reasoning [18]. Fuzzy inference is the process of developing the mapping from a given input to an output using fuzzy logic which then offers a base from which decisions can be made or patterns perceived. The classical logic has only two truth values, true or false, and so the process of inference is simplified as compared to fuzzy logic, where we have to be concerned not only with propositions but also with their truth values. Every FES has a fuzzy inference system that reasons using fuzzy logic membership functions, which refers to the degree to which the value of a particular attribute belongs to a set. The FES designed and employed in this paper can be generalized by means of a simple structure as shown in Figure 1. 

This illustrates the typical process flow as clear-cut stages for lucidity but in reality the process is not usually composed of such disjoined distinct steps and many of the stages, although present, are glazed over into one another [19, 20].

### 2.2. Model Development

The FES developed in this paper employs the Mamdani type fuzzy inference technique. This technique is performed in four steps:

- fuzzification of the input variables: done by the fuzzification module, which translates crisp inputs into fuzzy ones; that is, classical measurements are converted to fuzzy values through the use of linguistic variables;
- application of the Fuzzy operator and formation of rules for evaluation: done by a set of if-then fuzzy rule bases or knowledge bases, consisting of a set of conditioned fuzzy propositions;
- aggregation of the rule outputs: done by the fuzzy inference engine which has a specific inference method—here the Mamdani type. It applies fuzzy reasoning mechanisms to obtain outputs and carries out the computation using fuzzy rules;
- defuzzification: done by a defuzzification module which transforms fuzzy outputs back to crisp values.

### 2.3. Fuzzification

**Figure 2:**
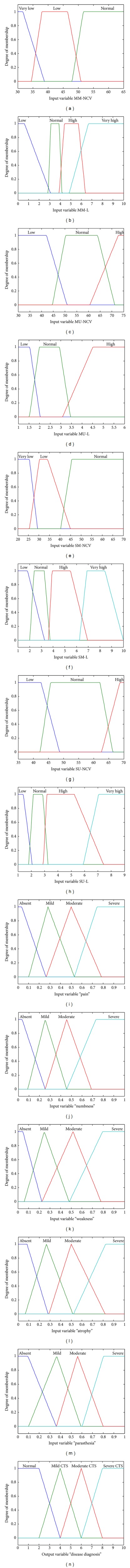
Membership function plots for the various input and output variables. (a) Motor median NCV. (b) Motor median latency. (c) Motor ulnar NCV. (d) Motor ulnar latency. (e) Sensory median NCV. (f) Sensory median latency. (g) Sensory ulnar NCV. (h) Sensory ulnar latency. (i) Pain. (j) Numbness. (k) Weakness. (l) Atrophy. (m) Parasthesia. (n) Output variable—disease diagnosis.

The fuzzifier converts the crisp inputs which are supplied to the system to fuzzy inputs and also determine the degree to which these inputs belong to each of the appropriate fuzzy sets. These fuzzy inputs are then used in the inference engine to generate fuzzy outputs. Eliciting knowledge from experts encounters numerous obstacles. The experts, in spite of being highly skilled in solving problems in their field, often feel difficulty in stating their knowledge in an orderly and logical manner or sometimes even in understanding their own decision making processes. For developing diagnostic tool for CTS, data is required that is capable of representing the disease as well as the severity of the disease. Basically the data consists of physical signs and symptoms of patients, medical reports, and so forth. By consulting the specialist and by analysing the data of the patients eight NCS values and five symptoms were finalized as the inputs for diagnosing the severity of the disease. The thirteen attributes which were considered here as the input variables for the diagnostic system were crucial and had to be considered for the diagnosis and the detection of severity of CTS. Fuzzy values were assigned for each of these input variables to get different fuzzy sets based on the expertise of the specialists and knowledge from the standard textbooks. These fuzzy mapping or membership functions can have a variety of shapes depending on how the expert relates different domain values to belief values. Triangular or trapezoidal shapes can simplify computation. The membership function parameters for the input variables and the membership function plots for the input variables and the output variable is shown in Table 2 and Figure 2, respectively.

#### 2.3.1. Rule Determination and Rule Evaluation

The basic requirement of rule-based systems is that the expert's knowledge and thinking patterns should be specified in an explicit manner. The development of such an expert system usually requires a domain expert, who knows how to solve the problem at hand but is not much informed or familiar with computer programming, and a knowledge engineer, well versed in the design of expert systems and computer technology involved but with little or no knowledge of the problem at hand. The set of rules in an FES is known as the rule base or knowledge base.

 Fuzzy rule-based systems, in addition to handling of uncertainties, also have several additional capabilities. Here approximate numerical values can be specified as fuzzy numbers. The performance of an FES mainly depends on its rule base so the optimization of the membership function distributions stored in the data base is the most important process. The rules in a Fuzzy Expert System are in the form following: If *x* is low and *y* is medium, then *z* is high, where *x* and *y* are input variables, *z* is an output variable, low is a membership function (fuzzy subset) defined on *x*, medium is a membership function defined on *y*, and high is a membership function defined on *z*. The antecedent or the preceding part (the rule's premise) describes the degree to which the rule applies, while the conclusion part (the rule's consequent) assigns a membership function to each of the output variables. If a fuzzy rule has more than one antecedent, the fuzzy operator AND or OR is used to obtain a single value that represents the result of the antecedent evaluation. Based on the descriptions of the input and output variables, 75 rules were constructed by selecting an item in each input and output variable box and one connection (AND). None was chosen as one of the variable qualities to exclude any of the variables from a given rule. The weight was specified to unity.

#### 2.3.2. Aggregation of the Rule Outputs

It is the process of the unification of the rules. The membership functions of all the rule consequents previously clipped during rule evaluation are taken and combined into a single fuzzy set. In this process a number of clipped consequent membership functions are changed into one fuzzy set for each output variable. The inference methodology used is the Mamdani inference method. In Mamdani inference method rules are of the following form.

*R*_*i*_: if *x*_1_ is *A*_*i*1_ and *⋯* and *x*_*r*_ is *A*_*ir*_ then *y* is *C*_*i*_ for *i* = 1, 2,…, *L*, where *L* is the number of rules, *x*_*j*_ (*j* = 1, 2,…, *r*) are the input variables, *y* is the output variable, and *A*_*ij*_ and *C*_*i*_ are fuzzy sets that are characterized by membership functions *A*_*ij*_(*x*_*j*_) and *C*_*i*_(*y*), respectively. The consequence of each rule is characterized by a fuzzy set *C*_*i*_. The final output of a Mamdani system is one or more arbitrarily complex fuzzy sets which usually need to be defuzzified.

#### 2.3.3. Defuzzification of the Output

Though the concept of fuzziness helps the rule evaluation during the intermediate steps, the final desired output for each variable is generally a single number, that is, a crisp value. However, the aggregate of a fuzzy set constitutes a range of output values, and so it must be defuzzified in order to resolve a single output value from the set. The defuzzification method used here was the centroid calculation, which returns the center of area under the curve. The defuzzified value was calculated based on the following equation:

(1) $$\begin{array}{c} d_{\text{CA}}\left(C\right)=\frac{\int_{-c}^{c}C\left(z\right)z\;dz}{\int_{-c}^{c}C\left(z\right)dz}, \end{array}$$

where *d*_CA_(*C*) is the defuzzified value and *C* is the membership function. Every rule was examined for a given set of input values using the AND operation and the rule which satisfied the operational logic was used to generate the output for the inference system. The output given by each rule was aggregated and then defuzzified using centroid calculation to generate a single output which was a single number representing the severity of CTS.

After setting up the fuzzy inference system (FIS) the next step was building the system with fuzzy logic controller (FLC) with Rule Viewer block. This implements the FIS with the Rule Viewer in simulink. Once we create the fuzzy system we can readily embed our system directly into a simulation and integrate it with the FIS. For the Mamdani FIS, the FLC block automatically generates a hierarchical block diagram representation of the FIS. This automatic model generation ability is called the *fuzzy wizard*. The block diagram representation only uses built-in Simulink blocks and, therefore, allows for efficient code generation. The FLC with rule viewer block is an extension of the fuzzy logic controller block. It allows us to visualize how rules are fired during simulation. We had also included a subsystem along with the FLC which consists of an embedded Matlab program and other blocks along with the display blocks. When the simulation is run the diagnosis appears as display along with its membership function.

## 3. Results 

### 3.1. Testing of the System

**Figure 3:**
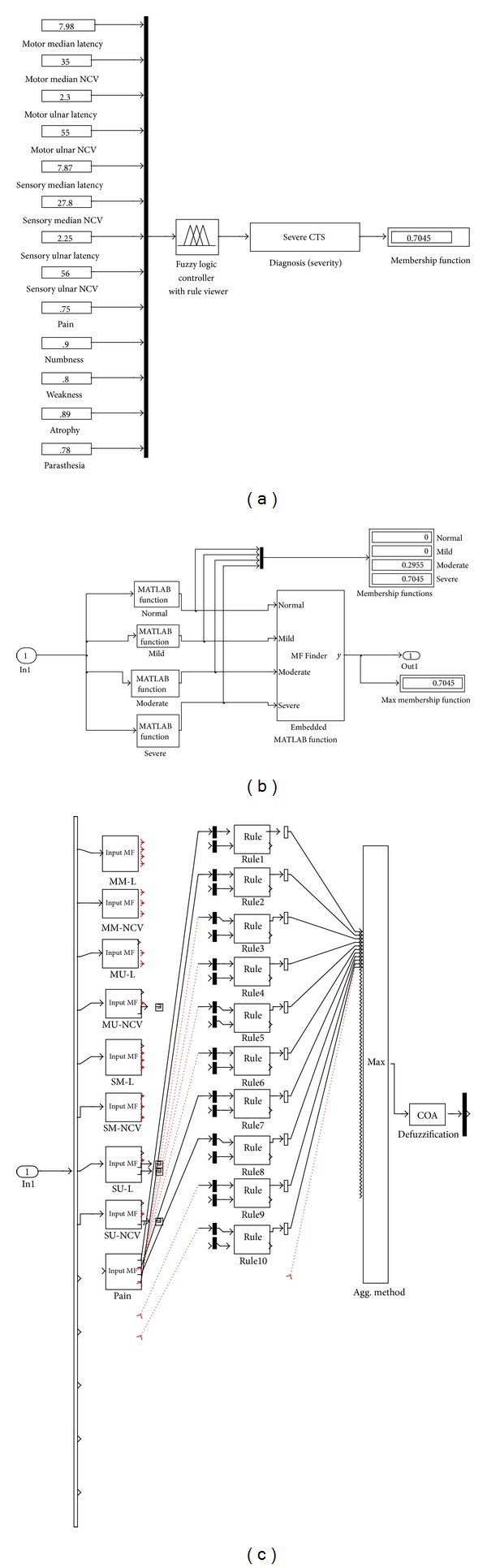
The Fuzzy Expert System developed in Simulink. (a) The FES with the FLC with rule viewer block; the subsystem block labeled diagnosis displays the result of the diagnosis whether it is normal, mild, moderate, or severe CTS; the display block labeled membership function gives the membership function of the diagnosis. (b) The subsystem diagnosis (severity) with an embedded MATLAB function, displays the membership functions of the output. (c) A portion of the FIS wizard block.

After the setup of the fuzzy inference system and its implementation in Simulink, the system was tested with various input values. The FES developed is shown in Figure 3. In the first part with the given inputs, the result is shown as “severe CTS” in the diagnosis (severity) block and the membership function shown is 0.7045 which means the diagnosis is severe CTS with a membership function 0.7045. The second part shows the Diagnosis (Severity) block; in that the display is showing that the output is having zero membership functions for “normal” and “mild”, membership function for “moderate” is 0.2955, and that for “severe” is 0.7045. So this block does a maximization and finally provides the main block with the output as “Severe CTS” and its membership function as equal to 0.7045. The third part shows a small part of the implementation of the FIS wizard since the entire model is too large.

Based on the rules the inference system calculated the severity of CTS by following AND connection and then defuzzification of the generated output using the centroid method. In fuzzy logic the truth of a statement is matter of degree so the AND connection performed a min operation. Based on the AND operation every rule was examined for a given set of input values and the rule which satisfied the operational logic was used to generate the output for the inference system. 

### 3.2. The Rule Viewer

**Figure 4:**
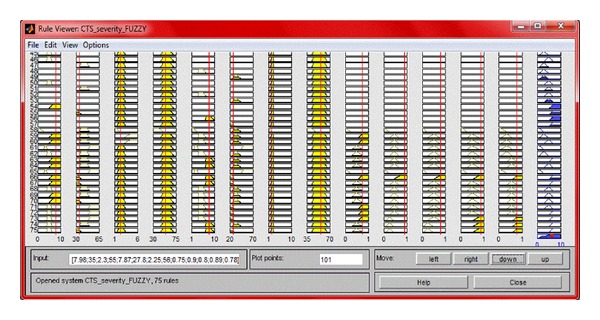
Rule viewer.

Based on these rules the roadmap of the whole FIS rule viewer is obtained as shown in Figure 4. Rule Viewer shows the active rules, the individual membership functions, and how they are influencing the results. It displays a guideline of the entire fuzzy inference process. In Figure 4 the fourteen plots shown represent the antecedents and consequent of each rule. Each rule consists of a row of plots, and each column gives the value of a particular variable. On the left of each row we can see the rule numbers displayed. 

The first thirteen columns of plots show the if part of each rule and the fourteenth column of plots shows the then part of each rule. The plots which are blank in the if part of any rule represent the depiction of “none” for the variable in the rule. The last plot in the fourteenth column of plots corresponds to the aggregates weighted decision for the given inference system and it depends upon the input values of the system. Though during the intermediate steps of rule evaluation we are dealing with fuzzy values, the final output for each variable is in a crisp form. So the aggregate of the fuzzy sets are defuzzified in order to resolve upon a single output value from the set. The defuzzification method used is the centroid method, and it returns the center of area under the curve which is displayed as a bold vertical line on this plot. On the topmost part above each column, the current values of each of the input variables are displayed. The variables and their current values are displayed on top of the columns. The Rule Viewer provides us with a visual display of the interpretation of the entire fuzzy inference process and it also expresses how the shape of certain membership functions determines the overall result. The Rule Viewer can be looked upon as a sort of microview of the FIS since it shows one computation at a time, in great detail. 

### 3.3. The Surface Viewer Plot

**Figure 5:**
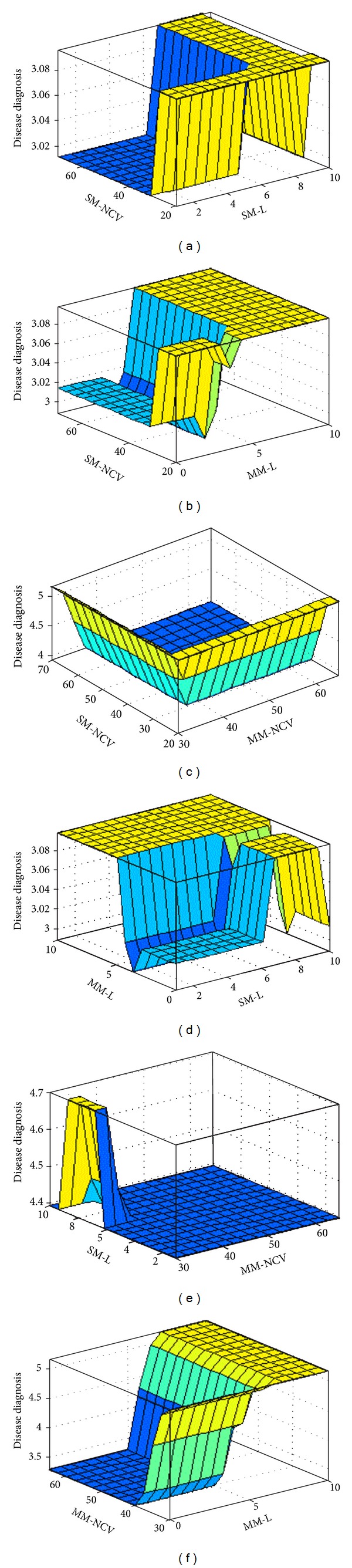
Surface Viewer plot of severity of CTS between different input attributes. (a) Sensory motor NCV and sensory motor latency (SM-NCV: SM-L). (b) Sensory motor NCV and motor median latency (SM-NCV: MM-L). (c) Sensory motor NCV and motor median NCV (SM-NCV: MM-NCV). (d) Motor median latency and sensory motor latency (MM-L: SM-L). (e) Sensory motor latency and motor median NCV (SM-L: MM-NCV). (f) Motor median NCV and motor median latency (MM-NCV: MM-L).

The surface viewer plot is shown in Figure 5. It renders a 3D surface from two input variables and the output of a FIS. It displays the dependency of the output on any one or two of the inputs; that is, it generates and plots an output surface map for the system. The Rule Viewer and the surface viewer are strictly read-only tools and cannot be used for editing. The Surface Viewer lets us select any two inputs and any one output for plotting.

The surface viewer clearly shows that the severity of CTS increases when both the motor and the sensory latency values increase. It also shows that when the motor or the sensory NCV value becomes very low the severity of the disease increases.

### 3.4. Accuracy of the System

Using the developed FES we tested the 135 NCS data which had normal, mild, moderate, and severe cases. The accuracy of the system was thus found out with the consultation of the expert; the FES showed a fairly good accuracy of 98.46% as shown in Table 3. It is also seen from Table 4 that the severe CTS stage has the highest sensitivity rate and specificity rate for the given FES diagnostic system. Sensitivity measures the proportion of actual positives which are correctly identified as such; that is, it relates to the test's ability to identify positive results while specificity measures the proportion of negatives which are correctly identified, that is, the ability of the test to identify negative results. The positive predictive value of a test shows how likely it is that a particular patient has the disease given that the test result is positive, and the negative predictive value gives the measure of how likely it is that the patient does not have the disease given that the test result is negative.

## 4. Conclusion

Diagnosis and management of diseases are indeed a difficult task that cannot be acquired from textbooks or classroom knowledge alone. It has to be acquired slowly through years of observation and experience. This is because most clinical scenarios have a vagueness varying in degree associated with them. During assessment the patients often describe their symptoms using superlatives such as “never, rarely, sometimes, often, most of the times, and always” and each specific symptom may also appear graded as “mild, moderate, or severe.” This emphasizes the reality that almost all the symptoms are experienced and described in a dissimilar manner by individual patients. Medical problems, therefore, cannot be generalized or analyzed using binary logic, that is, with a “yes” or a “no,” and an analytical program is required. Fuzzy logic, which has the ability of merging human heuristics into computer-assisted decision making, is the best solution to the problem.

This work was undertaken with an aim to design an expert system for the diagnosis of CTS and its severity using fuzzy logic which will be helpful for the patient to take proper curative measures before the severity increases. The results obtained from the system reveal that the diagnostic system is giving expected results and its efficacy has been endorsed by the specialist doctor in the field. The system developed was not meant to replace the specialist, yet it can be used to assist a general practitioner or specialist in diagnosing and predicting patient's condition. The rules given in the expert system actually replicate the type of decision making done in the trained mind of a specialist. 

In comparison to pertinency of fuzzy logic in medicine [21, 22] the concept is still new in the field of neurosciences. This was clearly highlighted from the fact that the contribution to the literature on fuzzy logic was much less from neurosciences [23] compared to other disciplines of medicine [24–27]. But the utility of fuzzy logic and other such techniques in various branches of neurosciences has been attaining popularity in the last decade. In our recent works [28, 29] we had shown how artificial neural networks and computer programs could be used successfully for the diagnosis of peripheral nerve disorders such as CTS and neuropathy.

Employing the use of computer-aided techniques in medical applications could reduce the cost, time, human expertise, and medical error. In the arena of medical diagnosis it acts as a powerful tool to help doctors to examine and model clinical data and make use of them for a number of medical applications. The significance of the developed FES lies in the fact that management of CTS depends fully on the severity of the disease and it requires the knowledge and experience of a specialist/neurologist to give a correct diagnosis regarding the severity of CTS, so the developed expert system enables the less experienced junior doctors to arrive at a better diagnosis as it keeps the expert knowledge in an intelligent system to be used efficiently by others. 

But the major drawback of these studies, which make use of the NCS data is the inherent shortcomings of the interpretation of the results, which include lack of standardization and absence of population-based reference intervals. Thus we conclude that studies involving the use of such a Fuzzy Expert System in providing diagnostic and predictive medical opinions are highly promising for the future. They can add value if embedded into the routine clinical consultations and used judiciously but can never completely replace the clinician.

## Figures and Tables

**Table 1:** The attributes of the nerve conduction study dataset.

Attribute no.	Attribute description	Attribute range	Mean	Standard deviation
1	Motor median latency (msec)	2–10	4.1	1.32
2	Motor median nerve conduction velocity (m/sec)	30–70	54.35	10.38
3	Motor ulnar latency (msec)	2–10	3.5	1.69
4	Motor ulnar nerve conduction velocity (m/sec)	30–70	53.76	8.9
5	Sensory median latency (msec)	2–10	3.6	1.25
6	Sensory median nerve conduction velocity (m/sec)	30–70	49.8	9.79
7	Sensory ulnar latency (msec)	2–10	3.3	1.36
8	Sensory ulnar nerve conduction velocity (m/sec)	30–70	52.76	8.67

**Table 2:** Membership function parameters for the input variables.

Input variable	Membership function	Parameters
Motor median latency	Low	[0.0185 0.0145 0.615 3.082]
	Normal	[2.87 3.108 3.9 4.17]
	High	[3.902 4.4 5.73 6.39]
	Very high	[4.85 6.68 10.3 11.4]

Motor median NCV	Very low	[15.8 24.9 31.69 38.8]
	Low	[34.53 38 46.5 50.9]
	Normal	[47.95 51.7 65.1 70.7]

Motor ulnar latency	Low	[0.01 0.9732 1.54 2.03]
	Normal	[1.52 1.99 2.94 3.457]
	High	[3.082 4.51 6.21 6.66]

Motor ulnar NCV	Low	[24.1 28.6 42.1 50.86]
	Normal	[44.63 50.28 63.91 71.19]
	High	[60.61 72.74 75.91 90.71]

Sensory median latency	Low	[0.3763 0.6076 1.828 3.274]
	Normal	[2.01 2.38 3.25 3.746]
	High	[3.61 3.92 5.481 6.95]
	Very high	[6.252 6.89 8.42 10]

Sensory median NCV	Very low	[15 19.5 25.5 29.37]
	Low	[25.5 30.3 34.1 44.89]
	Normal	[40.3 45.6 85.2 87.8]

Sensory ulnar latency	Low	[0.234 0.678 1.395 2.07]
	Normal	[1.8 2.15 2.86 3.26]
	High	[2.9 3.17 5.23 7.427]
	Very high	[5.905 7.06 9.59 13.2]

Sensory ulnar NCV	Low	[22.4 33.6 42.56 48.75]
	Normal	[42.24 45.74 62.12 66.37]
	High	[62.62 68.7 71.5 82.7]

Pain	Absent	[0.014 0.029 0.03611 0.264]
	Mild	[0.102 0.283 0.5304]
	Moderate	[0.266 0.5 0.7844]
	Severe	[0.5304 0.743 1.001 1.19]

Numbness	Absent	[0.0103 0.0132 0.0357 0.253]
	Mild	[0.0886 0.253 0.456]
	Moderate	[0.253 0.456 0.6892]
	Severe	[0.456 0.7315 1.04 1.36]

Weakness	Absent	[0.013 0.024 0.0463 0.2235]
	Mild	[0.0595 0.247 0.4802]
	Moderate	[0.226 0.5172 0.779]
	Severe	[0.483 0.795 1.04 1.36]

Atrophy	Absent	[0.0136 0.014 0.0833 0.2817]
	Mild	[0.0675 0.266 0.5146]
	Moderate	[0.29 0.5 0.8188]
	Severe	[0.522 0.8241 1.03 1.35]

Parasthesia	Absent	[0.036 0.04 0.09392 0.36]
	Mild	[0.1 0.364 0.5913]
	Moderate	[0.361 0.56 0.8003]
	Severe	[0.602 0.8029 1.05 1.37]

**Table 3:** Distribution of clinician's diagnosis (actual) against FES (predicted) diagnosis.

	Predicted diagnosis			
	Normal	Mild to moderate CTS	Severe CTS	Total
Actual diagnosis				
Normal	33	3	0	36
Mild to moderate CTS	2	53	1	56
Severe CTS	0	1	42	43

Total	35	57	43	Accuracy = 98.46%

**Table 4:** Diagnostics tests.

	Sensitivity	Specificity	Positive predictive value	Negative predictive value
Normal	94.28%	97%	91.66%	97.9%
Mild to moderate CTS	93%	97.4%	94.6%	95%
Severe CTS	97.67%	98.9%	97.7%	96.8%
